# Pandemic influenza A (H1N1v) infection in pediatric population: a multicenter study in a North-East area of Italy

**DOI:** 10.1186/1824-7288-37-24

**Published:** 2011-05-19

**Authors:** Liviana Da Dalt, Chiara Chillemi, Maria Elena Cavicchiolo, Silvia Bressan, Arianna Calistri, Giorgio Palù, Giorgio Perilongo

**Affiliations:** 1 Department of Pediatrics, University of Padua, Via Giustiniani 3, 35128 Padova, Italy; 2 Department of Histology, Microbiology and Medical Biotechnologies, University of Padua, Italy

## Abstract

**Background -:**

Data on clinical presentation, morbidity and mortality of 2009 pandemic influenza virus (H1N1v) in paediatric population are still emerging; most of the data so far available came from selected cohorts of children admitted to tertiary care paediatric hospitals.

**Methods -:**

An observational study involving all the 19 Divisions of Paediatrics of the Veneto Region was conducted with the aim of investigating into the demographic and clinical characteristics, the treatment, the outcome and the risk factors for disease severity of H1N1v infection occurring in children.

**Results -:**

Two hundred children, median age of 4.15 years (range 0-15) were enrolled from the last week of October till the first week of January 2010 for an overall hospitalization rate of 23/100.000. At least one underlying medical condition was found in 44% of patients. Fever and cough were the most frequent symptoms (93% and 65% respectively). 11 patients (6%) were admitted to a PICU and 5 (2.5%) required mechanical ventilation. Antiviral therapy was administered in 103 patients (51.5%) Death occurred in 2 patients (1%); both had severe prior medical conditions. Pre-existing neurologic diseases (OR 7.82; 95%CI: 1.15-53.34), the presence of hypoxemia (OR 10.47; 95%CI: 2.12-51.70) and anemia (Haemoglobin < 10 g/dL) (OR 14.15; 95%CI: 2.36-84.64) were risk factor for Intensive Care Unit admission.

**Conclusions -:**

This observational study in a given area of North-East Italy confirms the rather favourable prognosis of children with influenza A H1N1 (2009). Pre-existing conditions, and which is new, significant anemia, are risk factors for a complicated course.

## Introduction

Compared to seasonal influenza, H1N1 2009 pandemic influenza virus (H1N1v) had an increased infection rate among children and adolescents [1-3]. Even though the illness has been mostly acute and self limited, a variable spectrum of disease severity has been reported from mild illness to death. Children with underlying chronic disease are at increased risk for complications and death from influenza [4-10].

However, data on clinical presentation, morbidity and mortality of novel influenza A H1N1v in paediatric population are still emerging. In fact, during weeks 49 to 52 of 2010, influenza activity in the Northern Hemisphere still increased, in particular in Europe, with A(H1N1) and B viruses predominating [11]. The United Kingdom (U.K.) continues to report high rates of influenza. As February of 2011 approximately 25% of intensive care beds are occupied by influenza cases and 112 influenza-related deaths have been reported. 95 of 100 fatal cases from which the virus has been sub-typed have been H1N1 (2009) related and the other 5, influenza type B. The majority of severe and fatal cases have been between adolescent and adult population [11].

For all of these facts it is important to collect and critically analyze clinical information that may be useful for the correct management of patients in the near future.

As a matter of fact, there is a paucity in this field and those that exist are usually cohorts of children in tertiary care hospital [4,12-16], thus not describing the actual severity of illness in all children admitted to any hospital.

The first cases of confirmed infection with the H1N1v virus in Italy were documented in Autumn 2009. By October 2010, 5.582.000 illnesses, 938 hospitalizations (455 in Intensive Care Units, ICU) and 228 deaths were estimated in Italy [17]. In order to investigate the demographic and clinical characteristics, the treatment and the outcome of a large cohort of Italian children hospitalized because of the H1N1v Infection, we conducted an observational study in the Veneto region, a geographic area located in the North-East of Italy. The current study is also aimed to look into the risk factors for disease severity.

## Patients and Methods

In Italy there is a system of seasonal flu epidemic surveillance, Influnet. Family doctors and paediatricians, called "sentinel physicians", representing all Italian Regions, insert the influenza cases observed during the epidemic season in a website. They share a common operative protocol. Every week the National Institute of Health makes provision to process data [17].

### Geographic setting

The study was a retrospective observational study involving all the 19 Divisions of Paediatrics of the Veneto Region, included two Paediatric Tertiary care centres with their own Paediatric Intensive Care Units (PICU). This area has a population of 867.521 subjects aged less than 15 years of age, representing 19% of all inhabitants.

### Inclusion criteria

Eligible patients were all children under 15 years of age hospitalized in paediatric wards for H1N1v infection documented by testing combined nasal and throat swabs. All samples were sent to the Microbiology and Virology Division of the University Hospital of Padua for the research of H1N1v virus using reverse-transcriptase Real-Time polymerase-chain-reaction assay (RT-PCR) [18]. When required, the neuroaminidase (NA) gene of the viral isolates was also sequenced, by automated Applied Biosystems sequanator. Data collection started since the first week in which the virus was detected (October 2009) in a hospitalized child and extended through the last week the virus was detected (January 2010).

### Data collection

Demographic and clinical data for each study patient were collected using a standardized data collection form that included demographic information, influenza vaccination history, underlying medical conditions, clinical signs and symptoms, selected laboratory tests, radiographic findings, therapy, treatment course, complications and outcome in terms of duration of hospitalization, need for PICU, discharge or death.

All information was sent to the data-collection centre in Padua where a computerized database was created. The study was approved by the Research Ethics Committee of Padua Hospital and of the participating centres.

### Statistical analysis

Continuous variables were expressed as median and interquartile range (IQR) due to the non-parametric distribution; categorical variables were expressed as percentages. Comparison for continuous variables was performed by means of Mann Whitney U test for non-normally distributed data. For categorical data, the X2 test with Yates correction for 2 × 2 tables was used. We used p < 0.05 as a threshold for statistical significance.

A multiple logistic regression model was used to identify variables independently associated with the outcome variable, namely admission to PICU. A stepwise regression procedure was used in obtaining the final model.

## Results

**Figure 1:**
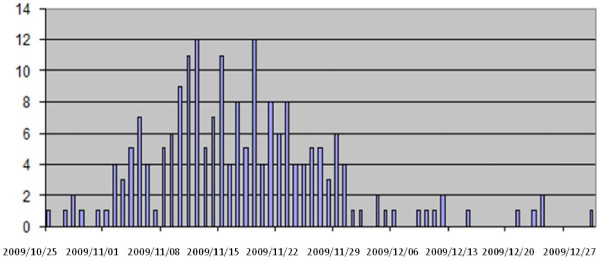
**Epidemiologic curve of hospitalization H1N1v virus infection (October 2009 and January 2010)**.

Two-hundred eligible children were enrolled during the study period. Fifty-seven (28.5%) were hospitalized in tertiary care centres. In the Veneto region the registered hospitalization for H1N1v influenza started in the last week of October with a peak in November 2009 and terminated in the first week of January 2010 at the time of the last reported case (Figure 1). The overall hospitalization rate was 23/100.000 children (age less than 15 years) and, more precisely, 20/100.000 in children less than 2 years of age, 40/100.000 in those aged 2 to 5 years and 27/100.000 in the older ones.

### Epidemiological and clinical characteristics

The epidemiological characteristics and the pre-existing conditions of the children of the cohort we studied are reported in table 1. The median age was 4.15 years with a slight male predominance; most of the children were Caucasian. Seventy-nine children (40%) had one or more pre-existing medical conditions, including chronic lung diseases (14%) and asthma (13%), hemoglobinopathies (14%), heart diseases (9%), neurologic disorders (28%), cancer (6%) and immunodeficiency (6%). Obesity was not represented in our sample as an underlying condition. The percentage of children suffering from chronic diseases was 70% in the two tertiary care centers and 27% in the remaining institutions. Three patients had received the H1N1v vaccine while seven the seasonal influenza vaccine.

**Table 1:** Demographic features and pre-existing conditions of the study population.

Demographic characteristics	
**Age (years)**	

Median	4.15

Range	0.8-7.5

**Age groups (years): N° of patients (%)**	

<2	73 (37)

2-5	42 (21)

5-18	85 (42)

**Male sex, N° (%)**	114 (57)

**Race, N° (%)**	

Caucasian	156 (78)

African	29 (14.5)

Asian	8 (4)

Others	7 (3.5)

**Pre-existing conditions, N°. (%)**	79/200 (40)

**Type of pre-existing conditions N°**	

Chronic lung disease	11/79 (14)

Asthma	10/79 (13)

Hemoglobinopathy	11/79 (14)

Heart disease	7/79 (9)

Neurological disease	22/79 (28)

Cancer	5/79 (6)

Immunosuppression	5/79 (6)

Other	8/79 (10)

**Vaccination status**	

H1N1 vaccine, N° (%)	3 (2)

Seasonal influenza vaccine, N° (%)	7 (4)

The median time from onset of symptoms to hospitalization was 1.3 days (range 0.5-4). Fever and cough were the two most frequent symptoms at admission (93% and 65% respectively - Table 2). Dyspnoea and wheezing were noted in 30% and 15.5% of the children respectively. Hypoxemia (sat 02 < 93%) at admission was present in 34 patients (17%). Gastrointestinal symptoms, such as vomiting (17.5%), diarrhoea (5%) or abdominal pain (5%) were also reported as well as aspecific symptoms such as fatigue and myalgia.

**Table 2:** Clinical, laboratory and instrumental findings of the study population.

**Clinical signs and symptoms**, **N° (%)**	
Fever	185 (93)

Cough	129 (65)

Dyspnoea	60 (30)

Wheezing	31 (15.5)

Chest pain	6 (3)

Rhinorrhea	69 (35)

Asthenia	98 (49)

Myalgia	7 (4)

Abdominal pain	11 (5)

Vomiting	35 (17.5)

Diarrhoea	10 (5)

Rash	3 (2)

Headache	2 (1)

Hypoxemia (sat 02 <93%), N° (%)	34 (17)

**Laboratory Findings**	

Leukocyte count (mm^3^) - median (range)	8480 (5447-11987)

Neutrophils (%) - median (range)	64 (39-77)

Haemoglobin (g/dl) - median (range)	11.9 (10.8-13.1)

Haemoglobin < 10 g/dL, N° (%)	28 (14.4)

Platelet count per mm^3 - ^median (range)	268.000 (201.500-355.000)

PCR (mg/l) - median (range)	8.1 (2.3-26.8)

Procalcitonin (μg/L) - median (range)	

Positive blood culture*- patients N°	4

Elevated aspartate aminotransferease, N°	7

**Chest Radiography**	

Pneumonia - N°/total N° (%)	107/132 (81.1)

Interstitial pneumonia, N° (%)	25 (24)

Lobar consolidation	79 (73.8)

Unilateral consolidation, N° (%)	44 (41.1)

Bilateral consolidation, N° (%)	20 (18.6)

Pleural effusion, N° (%)	5 (4)

*S. aureus, Candida glabrata, S. agalactiae, N. meningitidis

### Laboratory and instrumental findings

The laboratory and instrumental findings which were investigated are reported in Table 2.

Chest radiographs were suggestive of pneumonia in 104 patients, in most cases (76%) they showed lobar consolidation, unilateral or bilateral. In five cases pneumonia were associated with pleural effusion.

Laboratory confirmed co-infections were documented in nine children: four had a positive blood culture, respectively for *Candida glabrata*, *Streptoccocus agalactiae*, *Neisseria meningitidis*, and *Staphylococcus aureus *and 5 cases a positive serological test for recent *Mycoplasma pneumoniae *infection.

### Treatment

Antiviral therapy was administered in 103 patients (51.5%) after a median of 3 days (IQR 2-5) from the onset of symptoms; 46% of the children started antiviral therapy within 48 hours of the onset of symptoms. One-hundred-and-one children were treated with Oseltamivir and 2 with Zanamivir. In one case nebulized Zanamivir aqueous solution was administred for development of H1N1v viral mutation (H275Y), at the level of the NA gene, which conferred resistance to the initial treatment with Oseltamivir. The median duration of antiviral therapy was 4 days (IQR 4-5 days). Antiviral therapy was administered more frequently in patients with chronic diseases compared to healthy subjects (73% Vs. 37%, p < 0.0001) which explains why such therapy was administered more frequently in tertiary care centers compared with other pediatric institutions (84% versus 38%).

### Outcomes

Eleven patients (5.5%), ten with a pre-existing clinical conditions, were admitted to PICU; 6 (3%) required mechanical ventilation for a median duration of 7 days (IQR 3.75-26.5) and 1 extracorporeal membrane oxygenation (ECMO). The median age of patients admitted to PICU was 3.8 years (IQR 2.4-4.6). The median length of hospital stay was 4 days (IQR 3-6.5) while the median length of stay in ICU was 6.5 days (IQR 4-8).

Two children with severe pre-existing clinical conditions died (1%); one, a 3-year old girl affected by a complex congenital heart disease, died of combined respiratory and cardiac failure despite 10 days therapy with Oseltamivir and aggressive respiratory support including ECMO. The other patient, also a 3-year old girl, affected by Niemann Pick disease and suffering of an important hepatosplenomegaly, died of respiratory failure and superimposed Staphylococcus bacteraemia after 73 days of hospitalization.

As we can see on bivariate analysis (Table 3), that compared patients admitted to the PICU to those hospitalized in regular ward, clinical factors that resulted significantly more represented in the first group of children were: pre-existing clinical conditions and among them, neurological diseases, wheezing, dyspnoea, hypoxemia and a haemoglobin level less than 10 g/dL.

**Table 3:** Comparison between clinical characteristics of children admitted to the Intensive Care Unit and to the regular ward.

Variable	Admitted to ICU (11 pts)	Admitted to regular ward (189 pts)	P
**Age in years (range)**	3.8 (2.4-4.6)	4.3 (0.7-7.6)	0.834

**Sex, M/F**	7/4	107/82	0.885

**Pre-existing condition N° (%)**	10 (90.9)	69 (36.5)	0.0011

Asthma	0/11	10/189	0.94

Other chronic lung disease	0/11	11/189	0.88

Hemoglobinopathy	1/11	10/189	0.88

Heart disease	2/11	5/189	0.06

Neurologic disease	5/11	17/189	0.0011

Cancer	0/11	5/189	0.65

Immunosuppression	0/11	5/189	0.65

Other	2/11	6/189	0.09

**Clinical signs and symptoms**, **N° (%)**			

Fever	10 (91)	175 (92.6)	0.702

Cough	7 (63.6)	122 (64.5)	0.793

Dispnea	8 (72.7)	52 (27.5)	0.0045

Wheezing	5 (45.5)	26 (13.7)	0.0166

Hypoxemia at admission (Sat O2 <93%)	8 (72.7)	26 (13.7)	<0.0001

Chest pain	0	6 (3.2)	0.757

Rhinorrea	4 (36.4%)	65 (34.4)	0.847

Asthenia	6 (54.5%)	92 (48.6)	0.946

Myalgia	0	7 (3.7)	0.846

Abdominal Pain	0	11 (5.8)	0.886

Vomiting	3 (27.3%)	32 (17)	0.638

Diarrhea	0	10 (5.3)	0.940

**Laboratory Results (range)**			

Leukocyte--per mmc	5700 (4700-8380)	8600 (5590-12075)	0.1186

Neutrophils--per mmc	73 (47-77)	64 (39-77)	0.607

CRP--mg/L	28.5 (3-64)	7 (2.2-25.7)	0.137

Hb--g/dL	9.8 (8.4-11.1)	12 (11-13.2)	0.001

**Hb < 10 g/dL, N° (%)**	5/11 (45)	23/189 (12)	0.0046

**Chest Radiograph (132 patients)**			

Abnormal Chest X-rays, N° (%)	11/11 (100)	96/121 (79.3)	0.203

Interstitial/focal consolidation, N°	2/9	23/70	0.9143

Unilateral/Bilateral consolidation, N°	4/5	55/15	0.0752

Pleural effusion, N° (%)	1/11(9)	4/121 (3.3)	NS

**Therapy**			

Antiviral treatment, N° (%)	9 (81)	94 (50)	0.078

Days from onset of symptoms to initiation	5 (2-6.75)	3 (1.75-5)	0.203

Multivariate logistic-regression analysis showed that the following were independent risk factors for admission to the ICU: pre-existing neurologic diseases (OR 7.82; 95%CI: 1.15 to 53.34), the presence at admission of hypoxemia (OR 10.47; 95%CI: 2.12 to 51.70) and anemia (Haemoglobin < 10 g/dL) (OR 14.15; 95%CI: 2.36 to 84.64).

## Discussion

This series of hospitalized children infected with the influenza A H1N1v virus, one of the largest so far published, provides complementary data about the natural history and the clinical management of this disease in children, to the amount of information provided in such a short period of time since the first case documented on April 2009[19]. It includes patients all belonging to a well-defined area (the Veneto Region) and involves all paediatric institutions of any level. The hospitalization rate for children with influenza A H1N1v virus we documented, was equal to 23/100.000 with a peak in those aged between 2 and 5 years. It is difficult to compare these figures with the data available in the literature. In a series from California the hospitalization rate for all age groups was 2.8/100.000 ranging from 35.8/100.000 in infants 1 month old to 1.5/100.000 in patients aged 70 or older [7]. In the Argentina study, the corresponding figure was 20/100.000 in children less than 18 years [4]. Probably, differences in data collection and in the organization of the health system more than in the potential different clinical behaviour of the disease should be accounted to explain this geographic difference in the hospitalization rate. Certainly, influenza A H1N1v virus has affected more patients than seasonal influenza in a short time span worldwide [1]. Also in Italy, during the peak period in November 2009, the incidence of the H1N1v virus infection was 12.92/1000 compared to 8.23/1000 for 2007-2008 seasonal influenza in the same population [16].

In our series the median age of those children who were hospitalized was 4 years, a finding which is comparable to what most of series have so far reported [6,7,11,12,14,15,20]. Only the group from Argentina describes a much younger median age of those children who were hospitalized [4].

In the present series, the prevalence of children affected by pre-existing chronic conditions was relatively low - 39% - in comparison with other series which report values as high as 80% [6,7,10,12,15]. These pre-existing conditions were, in order of frequency: chronic pulmonary diseases, haemoglobinopathies and neurological disorders. The fact that this is an unselected series accounting also for those children admitted to non tertiary care centres, thus less likely to be suffering of chronic conditions, could be accounted for explaining the low prevalence of predisposing conditions we documented in our study population. In this study in fact the proportion of patients affected by chronic diseases was significantly higher in third level centers compared with the other provincial and local hospitals (70.2% Vs 27.3%, p < 0.0001). Furthermore, the low incidence in this study of cases of chronic asthma, obesity, clinical conditions which have been counted as a pre-existing conditions and highly represented in the series from America has contributed to this discordance [4,5]. Reading these data from a different point of view, the fact that slightly more than 60% of the children of this series were in a previous healthy status, underlines the potential severe clinical course which the disease may have in every children. In this regard it is worth highlighting the fact that one of the child of our cohort of young patients who ended-up in the PICU, was in a previously healthy status.

Despite these differences, the clinical, laboratory and radiographic manifestations of the H1N1v infection in this series were comparable to the ones reported by other studies.

As just said and described, the course of the H1N1v virus infection, despite generally very favourable, could be quite severe with even possible fatal outcomes. Severe pneumonia with respiratory distress syndrome, resulting in PICU admission, is the most common severe complication potentially bringing to death [4,5,9,10].

In this series only 5.5% of hospitalized children were admitted to PICU, rate which goes up to almost 20% if we considered only those children admitted to tertiary care centres. In terms of PICU admission rate these figures are surprisingly equal to the ones produced by the other paediatric series [4,6,14].

It is universally accepted that the presence of pre-existing conditions mainly neurological and cardiac disorders and asthma, are associated with a higher rate of admission to the PICU and death [4,7,9,11,12,14,15]. In this regard it is worth noticing that none of our patients who ended up in the PICU were affected by asthma. This is a remarkable difference from other series which report incidences of asthma among the pre-existing conditions leading children to the PICU up to 85%.

The high quality of asthma control within the general population could be one of the reason why we did not document this detrimental effect of asthma on the disease outcome [21]. In our cohort also anemia and hypoxemia resulted significant predictors of higher ICU admission rate. The rate of fatal outcomes in the cohort of children we studied is in the low range of the rates so far reported which varies from 1 to 5% [4,6,7,12,15]. Young age (less than 1 year) has been claimed as a risk factor for fatal outcome but the data has not been confirmed by our series as well as by others [4,6].

The impact of therapy and of an early intervention on the outcome of the infection has been extensively discussed in the different reports [22,23]. Half of the patients of this series were treated with neuroaminidase inhibitors including all the ones admitted to the PICU. At that time the principles which guided virus directed therapy were the following: to treat all children at risk; neonates and young infants, to rely on the clinical judgement for starting therapy in previously healthy children with uncomplicated diseases and ideally, to prolong treatment until complete virus clearance. However, from all the data so far produced no firm conclusions can be drawn on the role of this therapy and of an early intervention in reducing the length of hospital stay and/or the severity of the disease. The two patients of the present series who died were both affected by a sever pre-existing condition after having being treated for 10 and 73 days respectively with the appropriate therapy. Except for these two fatal outcomes, all other children had a full recovery which underlines once again the excellent prognosis of this viral infection.

It is worth mentioning the development, in a 3-year old severely immunocompromised patient, of resistance to oseltamivir, due to the appearance of H275Y mutation in the neuraminidase gene. A subsequent treatment with zanamivir was then able to clear the virus.

The main limits of this study were in the manner of data acquisition, since the collection was obtained by different clinicians, eventhough a uniformed report form was used and a good standardization of care exists in the Veneto Region and the fact that it relies on chart review.

## Conclusions

In summary, this quite large series of Italian children infected by the influenza A H1N1v virus collected by pulling together the epidemiological and clinical information coming from the various paediatric divisions of the Veneto region confirms that the prognosis is quite favorable for 11 previously healthy children hospitalized with influenza H1N1. However, serious complication may rarely occur among low-risk children, and the total disease burden is high.

These conclusions appear to be relevant especially considering that we are likely to face a new wave of H1N1v infection and data guiding towards the best management of patients are required.

## Competing interests

The authors declare that they have no competing interests.

## Authors' contributions

LDD and GP had partecipated in the development of the protocol and contributed to writing the manuscript. CC and MEC had the responsibility for protocol development, patient screeening and writing the manuscript. SB, AC and GP helped with the data analysis. All Authors had read and approved the final manuscript.
